# The Hospital World

**Published:** 1910-06-25

**Authors:** 


### The Hospital World.

### Elections and Appointments.

Lady Salvesen has become a patroness of the Edinburgh Royal Dispensary in place of the late Miss Mackenzie.

The Duke of Newcastle has been re-elected hon. president of the Retford Hospital and Dispensary, Sheffield.

The Earl of Radnor has been re-elected chairman, and Mr. J. A. Folliott vice-chairman, of the Salisbury and District Joint Isolation Hospital Committee.

Mr. James Arden has been re-appointed auditor to the Ulverston Cottage Hospital; and Mr. G. S. Miller, Mr. S. Pollitt, and Mr. Nelson Wearing have been elected to the committee.

The following Admission Committee for July, August, and September at the Royal Victoria Hospital, Belfast, has been appointed: Mr. J. C. Marsh (chairman), Mr. William Carson, and Miss Bruce.

The new directors of the Dundee Royal Infirmary have been appointed as follows: Mr. F. B. Sharp, Mr. William Rettie, Mr. H. K. Ogilvy, Mr. John Gibson, the Rev. A. B. Macaulay, and Dr. Peter Campbell.

The Right Hon. Earl Poulett has been appointed president; Mr. Haslock, Mr. F. J. Fry, and Mr. H. W. Paget Hoskyns, vice-presidents; Mr. R. H. Symons, treasurer of the Crewkerne Hospital, Somerset.

Sir Charles Seely, Bart., the Rev. Robert Holden, Mr. Frederick Acton, Mr. Frank E. Seely, and Mr. William Walters have been appointed to serve on the Ransom Memorial Joint Committee of the Nottingham General Hospital.

Sir W. Ogilvy Dalgleish, Bart., has been re-elected president of the Dundee Royal Infirmary. The following were elected vice-presidents: Lord Armitstead, Earl of Home, Mr. Walter Shepherd, Mr. R. G. Kennedy, and Mr. I. J. Weinberg.

The following officers of the Cobham Cottage Hospital have been re-elected: Mr. J. J. Morrish, chairman; the committee: Mrs. Allison, Mrs. W. Chamberlain, Mrs. Earle, Mrs. Trollope, and Mrs. Verrey, Col. Gordon Clark, Mr. S. A. Child, Mr. W. Drewett, Mr. R. L. Etherington, Mr. L. J. Longhurst, Mr. J. Vulliamy, and Mr. H. Waby; hon. treasurer, Mr. E. B. Nevinson; and hon. secretary, Mrs. Creswell.

The president and vice-presidents of the Bangor Cottage Hospital have been re-elected as follows: President, Lady Clanmorris; vice-presidents, Miss Connor, Mrs. F. R. Lepper, Mrs. Carson, the Rev. J. I. Peacocke, B.D., the Rev. John Waddell, B.A., the Rev. R. J. Morrell, the Rev. W. A. Hill, B.A., the Rev. James Stewart, the Rev. R. S. Lee, the Rev. H. Boyle, P.P., the Rev. S. S. Holmes, M.A., the Rev. J. A. W. Mulligan, B.A., the Rev. J. E. Hamilton, B.A., the Rev. S. W. Morrison, the Rev. Hugh Porter, and the Rev. W. J. M'Farland, B.A.

### Personalia.

We have to announce the death, at Burgh Heath, of Mr. George S. Saunders, formerly Librarian to St. Thomas's Hospital. In addition to the routine duties of Librarian, Mr. Saunders rearranged the books (over 7,000) in the Library, and drew up a card catalogue of authors' names. On his retirement the following resolution was passed by the Library Committee: "That the Library Committee has heard with much regret of the resignation of Mr. Saunders, the Librarian, and desires to place on record its appreciation of the services which he has rendered to the school during the eighteen years that he has been connected with it. It desires particularly to recognise his labour in connection with the organisation of the Library and the preparation of the catalogue, and his unvarying courtesy and kindness to his colleagues and to the students." In 1906 Mr. Saunders was elected a governor of the hospital. The funeral took place at Wandsworth Cemetery, and was attended by the Treasurer, representing St. Thomas's Hospital.

Ever since 1879 the Rev. Dr. Lippe has held the post of chaplain to the Aberdeen Royal Infirmary, and though his resignation was sent in in December last, he has only given up work since June 1. The thirty years of Dr. Lippe's ministry have seen a revolution in the hospitals of this country, and in this revolution the office of chaplain has shared. It has continued through good and through evil report; but that it has done so is due chiefly to the character of the holders of the office. No office in the hospital is less obtrusive to the public eye than that of chaplain, and it is fitting, therefore, that the only people who do know the quality of the work done, that is members of the board, should make it public when a long course of personal service is passed. The "affectionate regard" in which Dr. Lippe is held at Aberdeen has been the outward sign of a work the intrinsic value of which too often goes unsuspected. His successor is the Rev. Charles Stobie.

We take the following from the annual report of the Prince of Wales's General Hospital, Tottenham: "The Governors desire to place on record the great loss which the hospital has sustained by the death of their chairman, Sir Francis Cory-Wright. From the date of the re-organisation of the charity in 1899 this gentleman has presided over its affairs with marked ability, and although he was essentially one of the busy men of London, yet he devoted a considerable amount of time every year to the interests of the hospital, and it would be impossible to say how much the charity owes to his energy and generosity. Under his control the hospital has advanced steadily towards the important position it now occupies amongst the philanthropic institutions of the Metropolis, and the Governors are assured that they will receive the sympathy of their supporters in the trying circumstances they have had to contend with and their difficulty in securing a suitable successor."

